# A Multimodal Evaluation of an Emergency Department Electronic Tracking Board Utility Designed to Optimize Stretcher Utilization

**DOI:** 10.7759/cureus.11810

**Published:** 2020-11-30

**Authors:** Dirk Chisholm, Dongmei Wang, Thomas A Rich, Matthew Grabove, Kelli Sherlock, Eddy Lang

**Affiliations:** 1 Department of Medicine, University of Alberta, Edmonton, CAN; 2 Department of Emergency Medicine, University of Calgary, Calgary, CAN

**Keywords:** quality improvement, emergency department (ed) overcrowding

## Abstract

Objectives

The primary objective of this study was to evaluate the impact of an electronic tracking board feature encouraging staff to prompt optimal patient location on total stretcher time (TST) amongst patients moved to a chair in an internal emergency department (ED) waiting room. As a secondary objective, we also sought to identify facilitators and barriers to the tool’s use amongst the ED staff.

Methods

Using an administrative database, a retrospective cohort design was used to compare TST between visits where the tool was used and not used amongst patients relocated from initial assessment space to a chair over an 11.5 month period. A mixed-methods design was used to investigate facilitators and barriers to the tool’s use amongst the ED staff. Response proportions were used to report Likert scale questions; thematic analysis was used to code themes.

Results

A total of 56,852 patients met the inclusion criteria and were moved to a chair. The tool was used 4,301 times, with “OK for chairs” selected for 3,917/56,852 (6.9%) patients and “not OK for chairs” selected 384/56,852 (0.7%) times. Patient characteristics were similar between both groups. Median interquartile range (IQR) TST amongst patients moved to a chair via the prompt was shorter than when the prompt was not used (148.2 (112.6) mins vs 154.4 (115.4) mins, *p *= 0.005). A total of 125 questionnaires were completed; 95% of staff were aware of the tool and 70% agreed/strongly agreed the tool could improve ED flow. Commonly reported physician barriers to use were forgetting to use the tool; common nursing barriers were lack of chair space and increased workload.

Conclusions

Despite low function use, prompt use was associated with reduced TST amongst ED patients relocated to a chair.

## Introduction

While most Emergency Departments (EDs) discharge upwards of 80% of the patients they see, access block is a pervasive problem and is problematic, even during times when few admitted inpatients are being boarded in the ED [1]. This suggests that suboptimal utilization of ED stretchers is a contributor to ED access block. A study performed in a Canadian ED revealed that for patients with low-risk chest pain, 30% of stretcher time was unnecessary and patients could safely be transferred to chairs awaiting the results of a second cardiac biomarker result [2]. However, in instances where ED providers are available to assess new patients, it is not uncommon that they are unable to, given the lack of available stretcher space.

Optimal stretcher utilization is not a primary responsibility of the nursing staff or physicians in most EDs. For example, when patients are assessed or signed over from physician to physician at the end of the shift, there is rarely specific attention paid to whether the patients currently on a stretcher still need to occupy that stretcher until their treatment, investigations, or consultations reach completion.

In September of 2017, new functionality was embedded into the computerized physician order entry system used by four adult EDs in a major Canadian city. In the “location type” column of the patient tracking interface, a dropdown menu was installed with options, including “OK for chairs” and “not OK for chairs.” The selection of one of these options triggers the appearance of an icon adjacent to the patient’s name and current location to highlight their optimal location. The information transmitted between nursing and the ED physician is bi-directional and can be initiated by either, with the intention of prompting patient movement to their optimal location. To our knowledge, this is a novel tool, and no literature is available assessing the impact of electronic tracking board tools on ED stretcher use.

The primary objective of this study was to determine if the use of the "OK for chairs" functionality reduces total stretcher time amongst patients eventually moved to a chair. The secondary objective was to evaluate the effect of the tool’s use on ED length of stay (LOS). In order to explore the impact of moving patients from a stretcher to a chair on proxy measures of quality, the balancing measures of time to treatment order completion and time to diagnostic imaging completion were also compared between all patients moved, and not moved to a chair, regardless of the use of the prompting tool. As part of a qualitative analysis, we also sought to identify facilitators and barriers to the tool’s use amongst ED physicians and nursing staff.

## Materials and methods

A retrospective cohort design was used to examine the impact of the use of the prompt function (primary exposure) on the total time spent on a stretcher (primary outcome) for patients moved to a chair. Secondary outcomes included ED LOS, measured as time from triage to discharge. As part of the exploratory objectives, time to diagnostic imaging (time from order input to study) and time to treatment order completion (time from order input to completion) were compared between all patients who were moved to a chair versus not moved, regardless of whether the tool was used. 

Each use of the dropdown menu was recorded for each episode of use and was attributed to the nurse or physician who used the feature. These data were combined and stored on a secure server. All adult patients with a Canadian Triage Acuity System (CTAS) score of 2 to 5 who presented to any of the four adult EDs between September 1, 2017 and August 15, 2018, were relocated to a chair, and were subsequently discharged from the ED were included in the primary analysis [3]. Admitted patients were excluded since they were not the intended group for prompt use, and their total stretcher time/ED boarding time varied substantially as a function of hospital capacity, a variable that we were unable to capture. Moreover, patients located in the resuscitation/trauma or mental health areas were excluded. 

A mixed-methods study was used to investigate the facilitators and barriers to the tool’s use. A questionnaire examining attitudes, beliefs, use patterns, facilitators, and barriers were distributed electronically using the Research Electronic Data Capture (REDCap) tools (Vanderbilt University, Nashville, TN, USA) to a convenience sample of all local adult ED physicians and nursing staff (Appendix 1-2) [4]. 

Ethics approval was granted by the University of Calgary Conjoint Health Research Ethics Board for quantitative data; a waiver of consent was granted to use administrative data. In keeping with local best practices, an ethics screening tool was used to stratify risk associated with questionnaire data collection and was found to be low risk and not requiring full ethics review [5]. 

Analysis

Stata statistical software, version 13 (StataCorp LP, College Station, TX, USA) was used for all quantitative analyses. Descriptive statistics were used to present patient characteristics of age, CTAS score, sex, mode of arrival, triage scores, and the 10 most common Canadian Emergency Department Information System Presenting Complaints [6]. Median (interquartile range (IQR)) TST and ED LOS were compared between patients moved into a chair where the tool was used, and not used, using a Mann-Whitney U Test. Response proportions were used to report the questionnaire Likert scale items; thematic analysis was used to code themes in free text questionnaire fields using the NVivo 12 qualitative data analysis software (QSR International, Melbourne, Australia). 

## Results

Quantitative results

**Figure 1:**
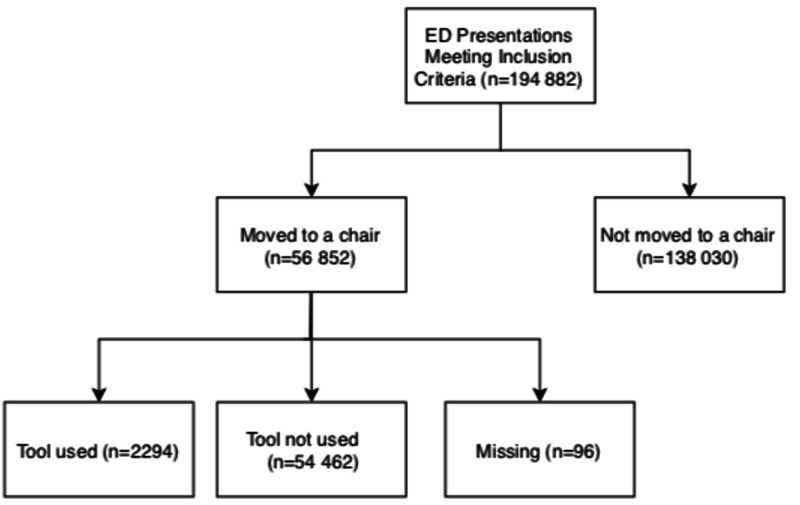
Final location of all patients meeting the inclusion criteria over the study period ED: emergency department

Of the 194,882 patient presentations to the ED meeting the inclusion criteria, 56,852 patients were relocated to a chair (Figure 1). Characteristics of the patients who were moved from a stretcher to a chair where “OK for chairs” was selected (n = 2,294) and those where the tool was not used (n = 54,462) are presented in Table 1. Mean age, sex, mode of arrival, CTAS scores, and the top 10 most common presenting complaints were similar between both groups. Amongst patients relocated from a stretcher to a chair, the total median stretcher time (IQR) was shorter in the group where the prompt was used (148.2 (112.6) mins vs. 154.4 (115.9) mins, p = 0.005). Median (IQR) ED LOS was similar between patients moved to a chair where "OK for chairs" was selected (263.6 (152.1) mins) compared to those where the tool was not used (265.3 (158.0) mins) (p = 0.22). 

**Table 1:** Characteristics of Patients Moved to a Chair Where "OK for Chairs" Was Used and Not Used CI: confidence interval; CTAS: Canadian Triage Acuity Scale; EMS: Emergency Medical Services

	OK for Chairs Used (n = 2,294)	OK for Chairs Not Used (n = 54,462)
Mean age, years (95% CI)	47.12 (46.4 - 47.9)	46.6 (46.4 - 46.7)
Sex		
Male (%)	981 (42.8)	22,281 (40.9)
Female (%)	1,313 (57.4)	32,181 (59.1)
Mode of Arrival		
EMS (%)	285 (12.4)	6,041 (11.1)
Not by EMS (%)	2,006 (87.5)	48,320 (88.7)
Missing (%)	43 (0.1)	101 (0.2)
CTAS		
2 (%)	787 (34.3)	17,445 (32.0)
3 (%)	1,003 (43.7)	25.473 (46.8)
4 (%)	435 (19.0)	9,838 (18.1)
5 (%)	69 (3.0)	1,706 (3.1)
Top Presenting Complaint		
#1	Abdominal pain, n = 387 (16.9%)	Abdominal pain, n = 10,516 (19.3%)
#2	Chest pain (cardiac type), n = 183 (8.0%)	Chest pain (cardiac type), n = 3,428 (6.3%)
#3	Flank pain, n = 91 (4.0%)	Flank pain, n = 2,836 (5.2%)
#4	Headache, n = 87 (3.8%)	Headache, n = 2,511 (4.6%)
#5	Cardiac type pain, n = 87 (3.8%)	Shortness of breath, n = 1,966 (3.6%)
#6	Shortness of breath, n = 80 (3.5%)	Chest pain (non-cardiac type), n = 1,964 (3.61%)
#7	Chest pain (non-cardiac type) n = 70 (3.1%)	Cough/congestion, n = 1,574 (2.9%)
#8	Cough/congestion n = 65 (2.8%)	Pregnancy issues < 20 weeks, n = 1,499 (2.8%)
#9	Lower extremity pain, n = 55 (2.4%)	Vomiting/nausea, n = 1,554 (2.9%)
#10	Dizziness, n = 53 (2.3%)	Cardiac type pain, n = 1,366 (2.5%)

Amongst all patients, regardless of prompt use, those moved to a chair had a similar median (IQR) time to medication administration (11.3 (22.5) minutes) compared to those not moved to a chair (9.1 (23.5) minutes). Similarly, patients moved to a chair had similar median (IQR) time to diagnostic imaging study (29.8 (47.8) minutes) compared to those not moved to a chair (25.1 (39.1) minutes).

**Figure 2:**
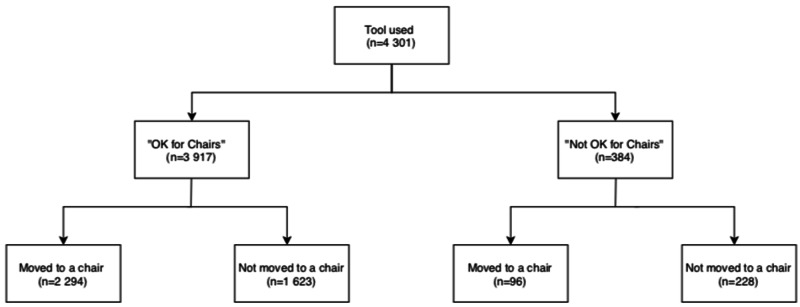
Proportion of patients moved to a chair after prompt use

Of all patients where "OK for chairs" was selected, 2,294/3,917 (58.6%) were relocated to a chair or recliner; 96/384 (25.0%) patients where “not OK for chairs” was selected were relocated to a chair (Figure 2). Physician use represented 86.8% (3,732/4,301) of all tool uses, with the remainder of uses by nursing staff (569/4,301, 13.2%). 

Questionnaire data

A total of 125 questionnaires were completed. The response rate was 8.5% (90/1200) for ED nurses and 17.5% (35/200) for ED physicians across all four adult EDs. Quantitative results are presented in Table 2. The majority, 119/125 (95.2%), were aware of the tool, while 45/118 (38.1%) reported “sometimes” using the tool and 49/119 (41.2%) reported patients were “sometimes” relocated after "OK for chairs" was used. Most respondents agreed (55/119; 56.2%) or strongly agreed (28/119; 19.3%) that the use of the tool could increase patient flow in the ED. Half of the respondents reported they very often use other methods to indicate patients who are suitable to be moved to a chair (59/118; 50.0%). Amongst nurses, 45/87 (51.7%) reported sometimes initiating conversations with physicians regarding relocating patients; 12/32 (37.5%) of physicians reported nurses rarely initiate conversations about relocating patients, while 12/32 (37.5%) reported nurses sometimes do so. 

**Table 2:** Electronic Questionnaire Responses from Physicians and Nursing Staff ED: emergency department

Question	Physicians	Nursing Staff
Are you aware of the "OK for chairs" function?		
Yes	32 (91.4%)	87 (96.7%)
No	3 (8.6%)	3 (3.3%)
How often do you use the "OK for chairs" function?		
Never	1 (3.1%)	8 (9.3%)
Rarely	13 (40.6%)	26 (30.2%)
Sometimes	10 (31.3%)	35 (40.7%)
Very often	8 (25.0%)	16 (18.6%)
Always	0	1 (1.2%)
How often is the patient relocated after an "OK for chairs" order is entered?		
Never	3 (9.4%)	0
Rarely	7 (21.9%)	12 (13.8%)
Sometimes	7 (21.9%)	42 (48.3%)
Very often	14 (43.8%)	29 (33.3%)
Always	1 (3.1%)	4 (4.6%)
To what extent do you agree that the use of the "OK for chairs" function could increase ED flow?		
Strongly agree	5 (15.6%)	23 (26.4%)
Agree	18 (56.3%)	37 (42.5%)
Undecided	8 (25.0%)	15 (17.2%)
Disagree	1 (3.1%)	12 (13.8%)
Strongly disagree	0	0
How often do you use other methods to indicate patients are able to be moved to a chair?		
Never	1 (3.1%)	2 (2.3%)
Rarely	2 (6.3%)	6 (7.0%)
Sometimes	11 (34.4%)	29 (33.7%)
Very often	14 (43.8%)	45 (52.3%)
Always	4 (12.5%)	4 (4.7%)
How often do you initiate a conversation with physicians about moving a patient to a chair?		
Never	N/A	1 (1.2%)
Rarely	N/A	6 (6.9%)
Sometimes	N/A	45 (51.7%)
Very often	N/A	29 (33.3%)
Always	N/A	6 (6.9%)
How often do nursing staff initiate a conversation about relocating a patient to a chair?		
Never	1 (3.1%)	N/A
Rarely	12 (37.5%)	N/A
Sometimes	12 (37.5%)	N/A
Very often	6 (18.8%)	N/A
Always	1 (3.1%)	N/A

As part of a thematic analysis, physicians reported the most common barrier to the tool’s use was forgetting to use the tool and a lack of perceived action by the nursing staff relocating patients. Common physician suggestions included making selection mandatory, creating a pop-up field, integrating location into the assessment orders, and creating less ambiguous location choices. Amongst nurses, commonly reported barriers to use were a lack of space/chairs to move patients to, the increased workload associated with patient turnover, and lack of nursing staff to take over care. Many nursing respondents reported the tool was already easy to use, but desired increased physician use of the tool. 

## Discussion

As part of the first study to assess the effect of a novel electronic tracking board utility to optimize the use of stretcher space, the use of the tool was associated with a statistically significant decrease in total stretcher time. Other characteristics, such as age, sex, mode of arrival, CTAS, and top presenting complaints were similar between patients moved using the prompt versus without the prompt (Table 1). Amongst all patients eventually relocated to a chair, the use of the prompt resulted in a median of 6.2 minutes of total stretcher time saved. Despite the modest reduction in stretcher time at the patient level, we would argue this reduction is clinically significant to ED operations. While we were not able to directly measure the impact of the use of the prompt on ED flow, one could theoretically extrapolate that if the prompt was used for all patients eventually moved to a chair over the 11.5 month study period, 5,891 hours of stretcher time would be saved across all sites, or 1,469 hours of stretcher time per site. 

Interestingly, only 58.6% of patients were actually moved to a chair when the prompt was used. While reasons for this are beyond the scope of this study, they may have included factors, such as lack of staffing or physical space in chair areas or patients being close to discharge when their location was prompted. 

Using the prompt did not result in a greater ED LOS. Similarly, being moved to a chair, regardless of prompt use, did not impact the proxy measures of quality of care of time to medication administration and time to diagnostic imaging. This would signal that moving a patient to a chair to await laboratory results, reassessment, or consultation does not negatively impact these domains of care.

Despite relatively low use of the tool, most staff reported being aware of the tool and believed the tool could be used to improve ED flow. Based on these qualitative data, future versions of the tool will consider prompting to increase the tool’s use, in addition to considering logistical considerations, such as chair space and nursing staffing needs. Other approaches to improve the proportion of eligible patients relocated to a chair, such as nurse-initiated protocols, should also be considered. 

Strengths and limitations

This is the first study to examine the effect of a novel, yet simple to implement tool designed to optimize ED stretcher use, and qualitatively examine facilitators and barriers to the tool’s use. Given the novelty of these findings, they could be used by other centers for consideration of implementing a similar prompt tool.

Our study also has limitations. The use of the tool was relatively low, and while it may be reasonable to postulate that a reduction in stretcher time could improve ED flow, we were not able to measure ED flow directly. Only patients discharged from the hospital were included, given they were the target population for use of the prompt. We were, therefore, unable to examine the effect of the prompt use for patients eventually admitted to the hospital. Patient characteristics were similar between those where the tool was used and not used. However, it is possible that unmeasured confounding variables could influence the relationship, given the univariate analysis. Moreover, 8.5% of the ED nurses and 17.5% of the ED physicians responded to the questionnaire; therefore, non-response bias could have impacted the qualitative results. We were unable to assess the impact of being relocated to a chair on patient experience in the ED. 

## Conclusions

While use was relatively low, the use of a novel, simple, and easily implemented electronic tracking board allowing staff to more effectively communicate which patients are appropriate to be relocated from a stretcher to a chair reduced total stretcher time. Further investigations are warranted to elucidate the effect of increased prompt use on ED flow. 

## Appendices

Appendix 1 

Physician Questionnaire 

Administrative Information: 

Date: 

Study ID: 

Site:

Questions: 

Are you aware of the "OK for chairs" function on Sunrise Clinical Manager (SCM)? 

Yes / No 

How often do you use the "OK for chairs" function? 

Never | Rarely | Sometimes | Very Often | Always 

How often do nursing staff initiate a conversation about relocating a patient to a chair, when they feel it is appropriate? 

Never | Rarely | Sometimes | Very Often | Always 

How often is the patient relocated after submitting an "OK for chairs" order? 

Never | Rarely | Sometimes | Very Often | Always 

To what extent do you agree that the use of the "OK for chairs" function could increase flow in the ED? 

Strongly Disagree | Disagree | Undecided | Agree | Strongly Agree                                                                                           

What would make it easier for you to use the "OK for chairs" function? 

What are the challenges you face using the function? 

Do you have any other comments? 

Appendix 2 

Nursing Staff Questionnaire 

Administrative Information: 

Date:

Study ID: 

Site:

Questions:

Are you aware of the "OK for chairs" function on Sunrise Clinical Manager (SCM)? 

Yes / No 

How often do you use the "OK for chairs" function? 

Never | Rarely | Sometimes | Very Often | Always 

How often do you initiate a conversation with physicians about moving a patient to a chair/waiting room when you feel it is indicated? 

Never | Rarely | Sometimes | Very Often | Always 

How often is the patient relocated after submitting an "OK for chairs" order? 

Never | Rarely | Sometimes | Very Often | Always 

To what extent do you agree that the use of the "OK for chairs" function could increase flow in the ED? 

Strongly Disagree | Disagree | Undecided | Agree | Strongly Agree 

What would make it easier for you to use the "OK for chairs" function? 

What are the challenges you face using the function? 

Do you have any other comments? 
